# Wide-Field Pixel Super-Resolution Colour Lensfree Microscope for Digital Pathology

**DOI:** 10.3389/fonc.2021.751223

**Published:** 2021-10-26

**Authors:** Guang Zeng, Jiahui He, Wenjian Qin

**Affiliations:** Shenzhen Institutes of Advanced Technology, Chinese Academy of Sciences, Shenzhen, China

**Keywords:** pixel super-resolution, digital pathology, wide field of view, colour holography, lensfree holography

## Abstract

Whole slide imaging enables scanning entire stained-glass slides with high resolution into digital images for the tissue morphology/molecular pathology assessment and analysis, which has increased in adoption for both clinical and research applications. As an alternative to conventional optical microscopy, lensfree holography imaging, which offers high resolution and a wide field of view (FOV) with digital focus, has been widely used in various types of biomedical imaging. However, accurate colour holographic imaging with pixel super-resolution reconstruction has remained a great challenge due to its coherent characteristic. In this work, we propose a wide-field pixel super-resolution colour lensfree microscopy by performing wavelength scanning pixel super-resolution and phase retrieval simultaneously on the three channels of red, green and blue (RGB), respectively. High-resolution RGB three-channel composite colour image is converted to the YUV space for separating the colour component and the brightness component, keeping the brightness component unchanged as well as enhancing the colour component through average filter, which not only eliminates the common rainbow artifacts of holographic colour reconstruction but also maintains the high-resolution details collected under different colour illuminations. We conducted experiments on the reconstruction of a USAF1951, stained lotus root and red bone marrow smear for performance evaluation of the spatial resolution and colour reconstruction with an imaging FOV >40 mm^2^.

## Introduction

Whole slide imaging (WSI), also known as digital pathology and virtual pathology, is a technique that captures stained pathological slides digitally at high speeds and high resolution for tissue morphology/molecular pathology assessment and analysis. Pathologists can zoom in and out on different sliding scales freely, as well as interpret lesions by quantitative image analysis (1). Brightfield illumination is usually used in WSI to observe stained pathological sliders under high magnification and high numerical aperture objectives. In traditional optical imaging, the relationship between its resolution and the field of view (FOV) is that the higher the resolution, the smaller the FOV. Therefore, the fact that traditional brightfield illumination with high-numerical aperture (NA) objective lens can only be used to acquire a small area of the pathological slide at a time for achieving high resolution. In order to capture the entire pathological slide, a considerable number of FOVs need to be collected through mechanical scanning, and finally, the image of whole slide is created by image stitching (2–4). The above digital process not only results in a complex system and prolonged data acquisition time but also requires high stability and reliability of the system. Besides, histopathological staining can help improve the colour contrast of cells and subcellular structures, which is useful for differentiating healthy cells from cancer cells. Therefore, accurate colour reconstruction is vital for pathological slide imaging (5–8).

**Figure 1:**
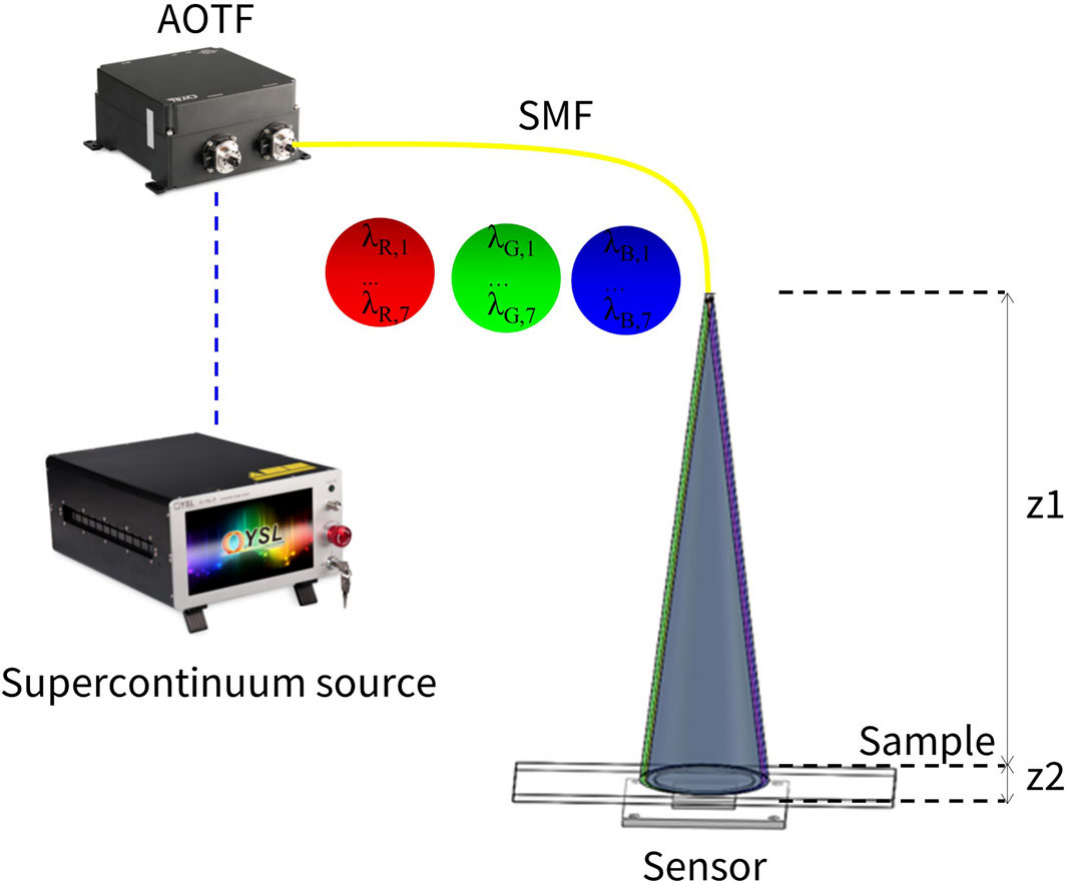
Schematic diagram of a wide-field pixel super-resolution colour lensfree microscope. The supercontinuum source outputs a continuum beam with a wavelength range of 410–2,100 nm. After being tuned by an acousto-optic tunable filter (AOTF), it is coupled into a single-mode fibre (SMF), and the SMF outputs wavelength scanning sequence of the three bands – R, G and B. The emitted light directly illuminates the sample without being collimated, and the image sensor records the light intensity signal.

Lensfree holography is an emerging microscopic imaging method (9–12). Its typical structure consists of a light source and an image sensor. The object to be imaged is placed between the light source and the image sensor, and there are no additional optical elements between the object and the light source as well as between the object and the image sensor. As shown in **Figure 1**, the distance z1 between the light source and sample is usually about 7–15 cm, and the distance z2 between the sample and sensor is about 100–1,000 µm (13). Lensfree holography has the advantage of decoupling the FOV and resolution to overcome the aforementioned challenge for WSI. In this imaging technique, the unit fringe magnification is achieved here due to the distance of z1 being much higher than z2. The theoretical resolution is equal to the pixel size of the image sensor, and thus, the photosensitive area of the image sensor serves as the imaging FOV (11). Under the same resolution, the FOV of lensfree holography is several times to hundreds of times than that of traditional optical microscopes (13–15). Nevertheless, there exists a limitation of spatial resolution due to the image sensor pixel size by manufacturing technology; pixel-based super-resolution methods have been developed to improve the resolution of lensfree holography, such as multiple sub-pixels shifts along the lateral direction of the light source, sample or image sensor etc. (16–19), by collecting multiple low-resolution images to synthesise one high-resolution image. All the methods mentioned above have high requirement on the stability of the system and usually require a large number of pictures for reconstruction calculation. Moreover, in order to address the limitation of image quality caused by twin images using purely pixel super-resolution, a series of phase recovery methods are also proposed to alleviate the interference of twin images. For instance, multi-frame images are collected through multiple wavelengths (10, 14, 20, 21) or multiple height methods (13–16, 22, 23), and the phase is iteratively restored using the idea of the Gerchberg–Saxton (GS) algorithm (24). The initial guess can be calculated by the transport-of-intensity equation (TIE) method to accelerate convergence (13, 15).

In recent years, multi-wavelength technology has also gained increased interest in lensfree holography to achieve pixel super-resolution. Ozcan et al. proposed to utilise very narrow wavelength bands with equally spaced wavelength scanning to achieve sub-pixel super-resolution (14). Despite the fact that the pixel super-resolution is achieved in the image reconstruction process, the phase retrieval is insufficient due to the narrow spectral band. Hence, image quality needs to be enhanced by combining multi-height phase retrieval. Zuochao et al. proposed a method to expand the wavelength scanning spectral band to obtain more wavelength diversity, which can achieve good phase recovery and pixel super-resolution only by wavelength scanning (10, 23). However, none of the above methods considered the colour reconstruction of the holographic image.

Accurate lensfree colour imaging has been proven to be challenging (21, 22, 25–27). The colour image reconstruction of the sample is not only affected by the intensity of the absorption of the illumination light in a specific wavelength band but also by the lack of phase information acquired by the image sensor (22). Iterative algorithm is able to eliminate a significant amount of colour artifacts caused by twin image and noise, yielding accurate phase information and improving the image quality and accuracy. This makes it possible to perform colour imaging on stained pathological slides. Most of the colour lensfree holography focuses on the phase retrieval of the holographic image, eliminating the interference of the twin image and colour artifacts on the reconstructed image. Currently, the colour imaging method focuses on the three wavelengths of red, green and blue (RGB) for separate illumination (28, 29), combined with multi-height phase retrieval or using colour image sensors to receive holograms of three wavelengths of RGB to create a colour image of the object. However, it does not consider the resolution enhancement processing of the colour image, making it unsuitable for the high-resolution imaging of a stained pathological slide.

This field has been greatly developed recently with a combination of colour reconstruction and pixel super-resolution. Although most of them focus on utilising complex motion-based pixel super-resolution and RGB colour reconstruction, colour artifacts cannot be eliminated effectively; meanwhile, the system is complex and time-consuming (22, 28). For instance, Ozcan et al. proposed a method of collecting low-resolution RGB holograms for colour reconstruction, which transferred the low- resolution colour imaging from the RGB colour space to the YUV colour space. After the conversion, the U and V colour components are averaged, and the Y bright component is replaced with a green wavelength pixel super-resolution image (22). Therefore, the final output removes the rainbow-like artifacts in the colour image by translating the YUV colour space back to the RGB colour space. However, the spectral absorption properties of different substances vary greatly (21). Hence, applying the above method, purely using a single green wavelength for pixel super-resolution, cannot satisfy the requirements of stained pathological slide imaging, which causes the loss of some important information.

In this paper, we propose a wide-field pixel super-resolution colour lensfree microscopy by performing wavelength scanning pixel super-resolution and phase retrieval simultaneously on the three channels of R, G and B in a relatively large spectral range. To alleviate rainbow-like artifacts superposed by twin-image noise and coherent illumination characteristic, the RGB colour space is converted into the YUV colour space. The U and V components are augmented by the average filter, respectively, while the Y component remains unchanged, and finally, the updated YUV is transferred back to the RGB space. Using this approach, the common rainbow artifacts of holographic colour reconstruction are significantly eliminated, as well as the high-resolution details collected under different colour illumination are preserved.

## Material and Methods

The objective of this work is to develop a novel lensfree microscopy imaging method for digital pathology by wide-field pixel super-resolution along with high signal-to-noise ratio (SNR) colour reconstruction. This approach provides a large FOV and is reliable, fast and cost-effective compared to existing WSI techniques without mechanical scanning.

For the lensfree holographic imaging, the object is illuminated by a multi-wavelength {λ*_k_*} sequence. The object function at a certain wavelength is *o_k_* (*x*, *y*). Because of the lensless structure, the incident light can be regarded as a plane wave. For simplicity, it is described as ${\text{e}}^{j2\pi \left(f_{x,k}\;\cdot x+f_{y,k}\cdot y\right)}$ , the interaction between the plane wave and the object is denoted as $o_{k}\left(x,y\right)\cdot {\text{e}}^{j2\pi \left(f_{x,k}\cdot x+f_{y,k}\cdot y\right)}$ , and its Fourier transform is *O_k_* (*f_x_* – *f*_*x,k*_, *f_y_* – *f*_*y,k*_), in which


$$f_{x,k}=n_{k}sin\theta_{k}cos\varphi_{k}/\lambda_{k}$$

$$f_{y,k}=n_{k}sin\theta_{k}sin\varphi_{k}/\lambda_{k}$$

(*θ_k_*, *φ_k_*) represent the angle of incidence, and *n_k_* is the refractive index between the sample and the image sensor (14). It can be seen that the change of the wavelength actually corresponds to the displacement of the object in the frequency space, i.e., the more the wavelength, the more frequency information collected, which lays the foundation for implementing pixel super-resolution. The phase retrieval is similar to the iterative reconstruction of the Gerchberg–Saxton (GS) algorithm method (24), and the acquired real image is used to continuously correct the image obtained by the iterative calculation to achieve the phase retrieval.

### Experimental Set-Up

Our experimental set-up is schematically demonstrated in **Figure 1**, a supercontinuum laser source (YSL Photonics, SC-PRO-M, wavelength range: 410~2,400 nm, visible light power more than 2W, seed source pulse duration ~6 ps) passes through an acousto-optic tunable filter (YSL Photonics, AOTF-VIS, wavelength range 400~650 nm, bandwidth 2~7 nm). The output of the acousto-optic tunable filter (AOTF) is used as the illumination light source. The AOTF supported the simultaneous output of eight channels, and each channel has a different wavelength. All wavelength channels could be coupled to the same single-mode fibre. We directly use the light output from the single-mode fibre to illuminate the sample and keep the distance (z1) from the fibre exit to the sample by about 5~10 cm. The image sensor uses a board camera (HIKROBOT, MV-CB120-10UM, pixel size 1.85 µm), with the distance between the sample and the image sensor (z2) being smaller than 2 mm. The image sensor sequentially records the hologram of each wavelength. The wavelengths of the three RGB channels are selected as R: 606~642 nm, G: 512~548 nm and B: 452~488 nm. In each channel, the interval of each wavelength is 6 nm.

Since there exists a certain deviation between the actual wavelength output by the AOTF and the nominal wavelength, in order to guarantee the quality of the experiment, we use a spectrometer (Ocean Optics, HR4000, detection range 200–1100 nm, optical resolution 0.75 nm, SNR 300:1) to measure the output wavelength of AOTF, and the measured value represents the standard wavelength for experiment conduction.

### Multi-Wavelength Scanning for Low-Resolution Hologram Acquisition

In order to colour holographic imaging, multi-wavelength scanning including three basic colours (blue, green and red) is commonly used to capture holograms for colour reconstruction. In this work, a sample is scanned in total of three wavebands, in which each waveband has seven wavelengths, with a total of 21 low-resolution holograms being collected. The collection method of each low-resolution hologram following the same acquisition criteria is as follows:

Firstly, the output single wavelength λ_k_ of the AOTF is set at a time, and the output light is coupled into the single-mode fibre. Secondly, the light is emitted from the optical fibre directly without collimating, which propagates through free space and then presents as incident light on the surface of sample. Finally, after the incident light has undergone amplitude and phase modulation by the sample, the emitted light leaving from the sample is recorded by the image sensor. Since z1 >> z2, the light wave incident on the sample surface can be approximated as a plane wave.

### Pixel Super-Resolution and Phase Retrieval on Multi-Wavebands

**Figure 2:**
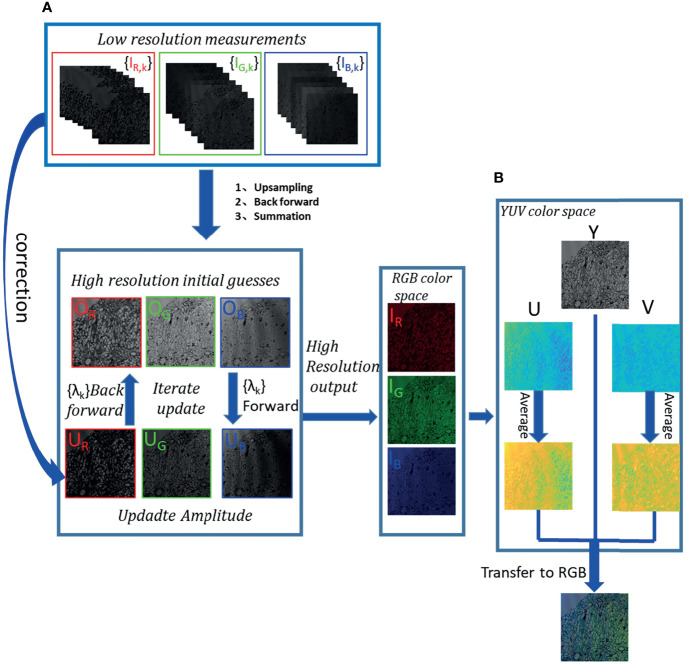
Flow chart of multi-wavelength scanning pixel super-resolution and colour reconstruction. **(A)** After low-resolution measurement hologram stacks are up-sampled, backward-propagated and summed, a high-resolution initial guess is generated. The high-resolution object is iterated forward and backward in a sequence of wavelengths between the object surface and the image sensor plane. The measurement hologram is used to correct the wavefront amplitude of the image sensor plane, which is calculated by the forward propagation from the object plane to the image sensor plane. The object wavefront is updated by the backpropagation calculation from the image sensor plane to the object plane. And then, the phase relationship is used to correct the phase. Finally, the phase retrieval high-resolution reconstructed images of R, G and B channels are obtained. **(B)** A high-resolution RGB image from the RGB colour space to the YUV colour space is converted. With U and V components being average-filtered, respectively, the Y component remains unchanged. Then, the updated U and V are converted into the RGB space together with Y to generate a high-resolution colour reconstruction image that eliminates rainbow-like artifacts.

In colour holographic imaging, multi-wavelength scanning not only can be utilised to reconstruct the captured hologram by the three primary R, B and G wavelengths, respectively. It can also retrieve the phase profile (10). According to the theory of multi-wavelength-based phase retrieval, the different hologram amplitudes are crucial for phase retrieval calculation, which is acquired by different wavelength illuminations. Thus, the accurate reconstruction of phase images relies on high-SNR hologram acquisition. To overcome the limitation of pixel resolution caused by Complementary Metal Oxide Semiconductor (CMOS) sensor technology, multi-wavelength scanning also enables pixel super-resolution on low-SNR recorded holograms (10, 23). Therefore, the phase estimation and pixel super-resolution reconstruction could be conducted at the same time by multi-wavelength scanning technology. The details of phase retrieval and pixel super-resolution are illustrated in **Figure 2**, and the specific implementation is composed of the following steps:

**Step 1:** As depicted in **Figure 2A**, the multiple under-sampled low-resolution measurement holograms collected in the waveband are labelled as {*I_C,k_*}, and the wavelengths are labelled as {*λ_C,k_*} (*C* = {*R*, *G*, *B*}, *k* = 1,2,3,4,5,6,7).
**Step 2:** Previous reports (10, 14) point out that with the advantage of wavelength diversity, the high-resolution initial guess for the R/G/B-channel can be figured out. Under the condition that the up-sampling rate is set to 4, the low-resolution measurement holograms collected under the illumination of various wavelengths are up-sampled and named as {*I_C,k_*}*_upsample_*. After the {*I_C,k_*}*_upsample_* is transmitted backwards to the object surface by angular spectrum methods to obtain the corresponding object function, a good initial guess could be obtained by summing up all the object functions and then doing average filtering. The initial guess is recorded as *O_C_*.
**Step3:** *O_C_* propagates from the object plane to the image sensor surface at the wavelength *λ_C,k_* to obtain *U_C,k_*. The amplitude of *U_C,k_* is corrected by the low-resolution measurement hologram *I_C,k_* collected at the wavelength of *λ_C,k_*, and *U_C,k_* is updated.
**Step4:** The updated *U_C,k_* propagates back to the object plane at the wavelength *λ_C,k_*, and an updated *O_C_* is obtained.
**Step5:** Before *O_C_* propagates with *λ*_*C,k* + 1_, it is necessary to correct the phase with a phase relationship (30), while the amplitude remains unchanged and then updates the *O_C_* again. *O_C_* repeats steps 3 and 4 with wavelength *λ*_*C,k* + 1_, completing an iteration until all wavelengths in this band are being used and generally iterating 5–20 times. The final high-resolution value *O_C_* for pixel super-resolution and phase retrieval is obtained.

### RGB to YUV Space Conversion and Enhancement for Accurate Colour Reconstruction

Although reconstruction artifacts of in-line holography by twin image have been mitigated based on the multi-wavelength phase retrieval method, the rainbow-like artifacts manifested as different frequency stripes still cannot be avoided in forming into colour imaging. To solve the above issue, the brightness component of an RGB image is separated from its colour components, which converts the RGB image to the YUV space. In the YUV space, the brightness component characterising the details and the overall outline of the image are kept unchanged, and applying average filtering on the U, the V colour component for rainbow-like-artifact elimination (22).

The details of colour reconstruction can be summarised as follows:

(*i*): Obtain the high-resolution *O_R_*, *O_G_* and *O_B_* intensity images of the channels *R*, *G*, and *B* by the abovementioned method.
(*ii*): Combine intensity images into channels *R*, *G* and *B* of the RGB colour space to obtain the corresponding colour images, recorded as *I_R_*, *I_G_* and *I_B_*, respectively.
(*iii*): Convert *I_R_*, *I_G_* and *I_B_* to the YUV space to get *Y*, *U* and *V* components (22). *Y* is the brightness data, which remain unchanged, and *U* and *V* colour components are respectively average-filtered to obtain *U_ave_* and *V_ave_*.
(*iv*): The final accurate colour image is created by transforming *Y*, *U_ave_* and *V_ave_* back to the RGB space.

## Results

### Pixel Super-Resolution by Multi-Wavelength Scanning

**Figure 3:**
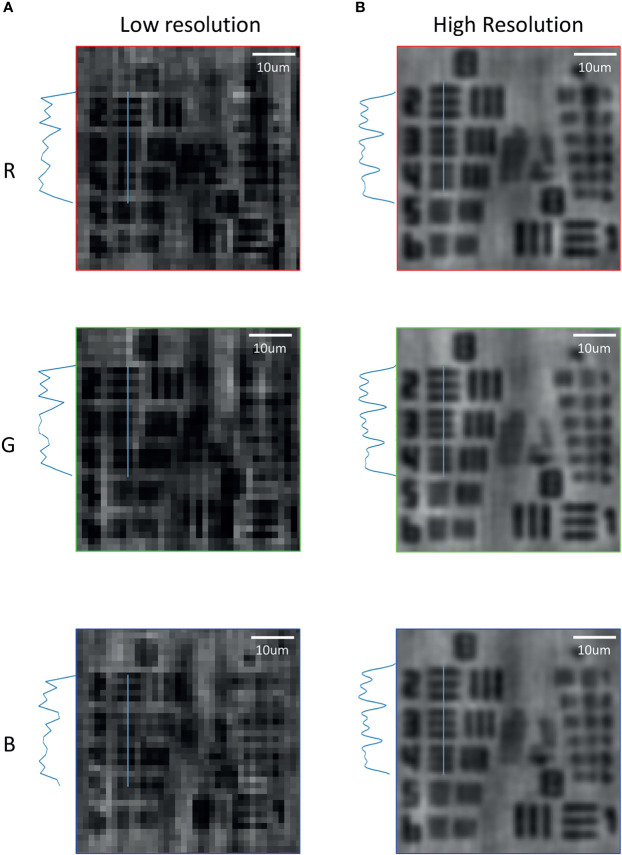
Comparison of the pixel super-resolution and low resolution in the bands of R, G and B on the USAF1951 resolution test chart. **(A)** Low-resolution reconstructed images in R, G and B bands. **(B)** High-resolution reconstructed images in R, G and B bands by the wavelength scanning method.

In order to quantify the spatial resolution performance of multi-wavelength scanning pixel super-resolution, the USAF1951 resolution test chart was imaged by placing on the image sensor and illuminated with a wavelength interval of 6 nm in each RGB channel (the wavelength range of each channel was R: 606~642 nm, G: 512~548 nm and B: 452~488 nm). Multiple low-resolution images obtained in each channel were synthesised into one high-resolution image. Then, the hologram is reconstructed at 624, 530 and 470 nm to obtain low-resolution reconstructed images. For comparison, the high- and low-resolution images of channel RGB were carried out, respectively. As shown in **Figure 3**, the [group 8, element 2, 1.74 µm] can be distinguished on the low-resolution reconstructed image of R, G and B channels, respectively. While after pixel super-resolution, [group 8, element 4, 1.38 µm] could be resolved on high-resolution reconstruction in the three R, G and B channels, respectively. It is noted that low-resolution images have lots of background noise, while better contrast was achieved on high-resolution images with clear backgrounds.

### Performance of Stained Samples Under Multi-Wavelength Illumination

**Figure 4:**
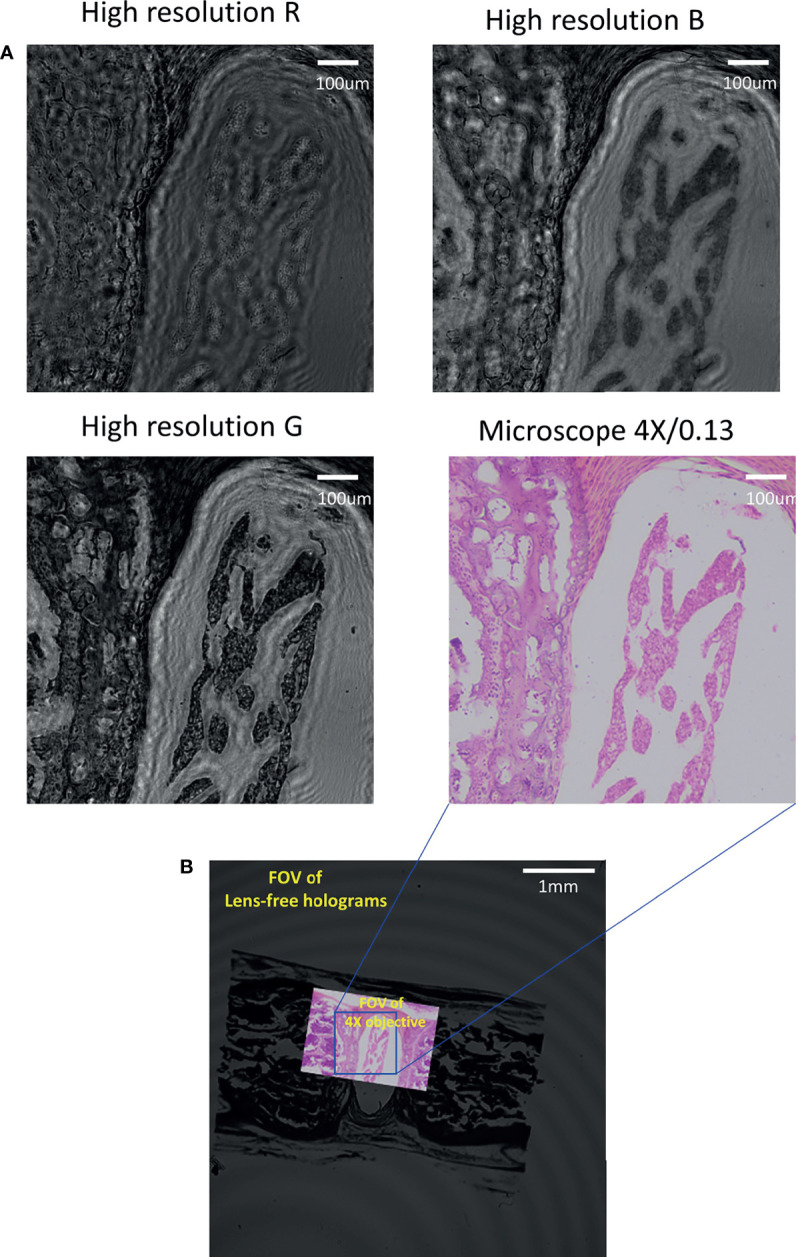
Comparing high-resolution images in R, G and B channels of red marrow smears with traditional bright-field microscopy. **(A)** The high-resolution images of each band were compared through the image acquired by the microscope (4X/0.13). **(B)** FOV comparison between the lensfree microscope and the traditional bright-field microscope.

To investigate the effect of different wavelengths for colour reconstruction, which is important for digital pathology diagnosis, we conducted experiments on a red bone marrow smear sample, which was irradiated alternately by the three wavebands of R, G and B. In our experiments, the low-resolution images were merged by low-resolution holograms by three illumination wavelengths (λ = 470,530, and 624 nm) directly, and high-resolution images were reconstructed by the multi-wavelength scanning technique with 21 low-resolution holograms. As demonstrated in **Figure 4**, it was noted that structure details of the three channels were quite different. Being observed under a microscope, the red bone marrow smear was reddish to purple; in other words, the transmittance of red and blue waves was predominant that it was detected by an image sensor. Particularly, the transmittance of red was higher than that of blue. **Figure 4A** showed that there was much fewer information on channels R and B of the holographic reconstruction image, which has more blank without object areas that meant that the corresponding transmittances of red and blue were high. Due to the strong absorption of green light, more features could be seen in the holographic reconstruction image of the corresponding Channel G. Hence, the result proved that under the illumination of different wavelengths of light, the reconstructed image was embodied by different details. As a result, the quality of reconstruction is beneficial from multi-wavelengths.

Moreover, the lensfree microscope shown in **Figure 4B** has much large FOV (>40 mm^2^) in comparison with 4 × microscope objective (0.13 NA), approximately 15 times more than the traditional bright-field microscope.

### Comparison Results of Colour Lensfree Imaging Reconstruction

**Figure 5:**
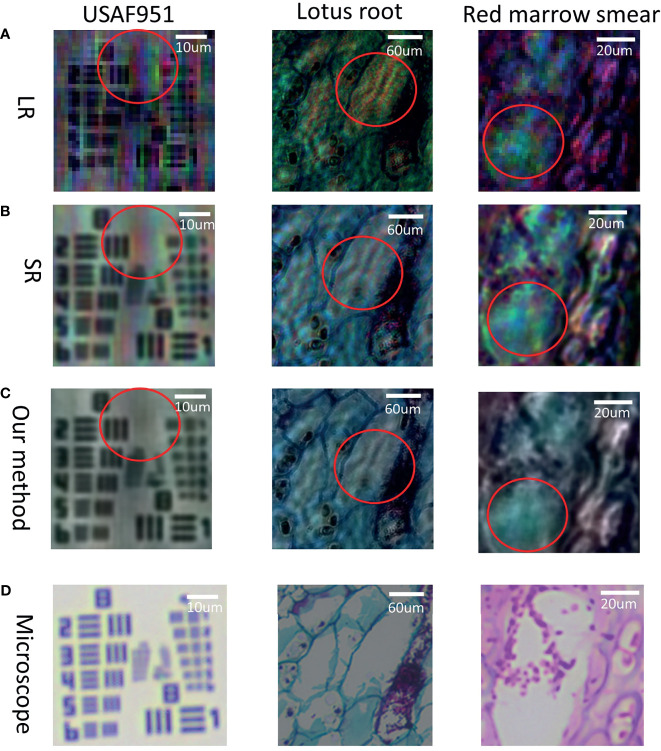
Comparison results of the resolution chart, the lotus root cross section, and the red marrow smear sample in different colour reconstruction modes along with the images taken under the microscope. **(A)** Low-resolution colour image without pixel super-resolution; **(B)** Colour image using wavelength scanning pixel super-resolution and phase retrieval; **(C)** Colour-reconstructed image proposed in this paper; **(D)** Colour images taken by microscope.

To compare the performance of colour reconstruction for lensfree holographic imaging, the multi-wavelength scanning technique for pixel super-resolution and phase retrieval were simultaneously used to reconstruct the high-resolution colour image of USAF1951, the cross section of the lotus root and the red marrow smear sample. **Figure 5** illustrated the comparison colour reconstruction results of different methods on the three samples. The first row is colour reconstruction by low-resolution holograms directly. The second row is reconstructed by the high-resolution holograms only (without colour enhancement). The third row is reconstructed by our proposed method. The bottom row images are obtained by the microscope. From the comparison results in **Figure 5**, it is clearly seen that the experimental results obtained by our method are more obvious in rainbow artifact removal than the other two holography methods. Consequently, the colour reconstruction performance of our method is better to support our conclusion.

## Discussion

**Figure 6:**
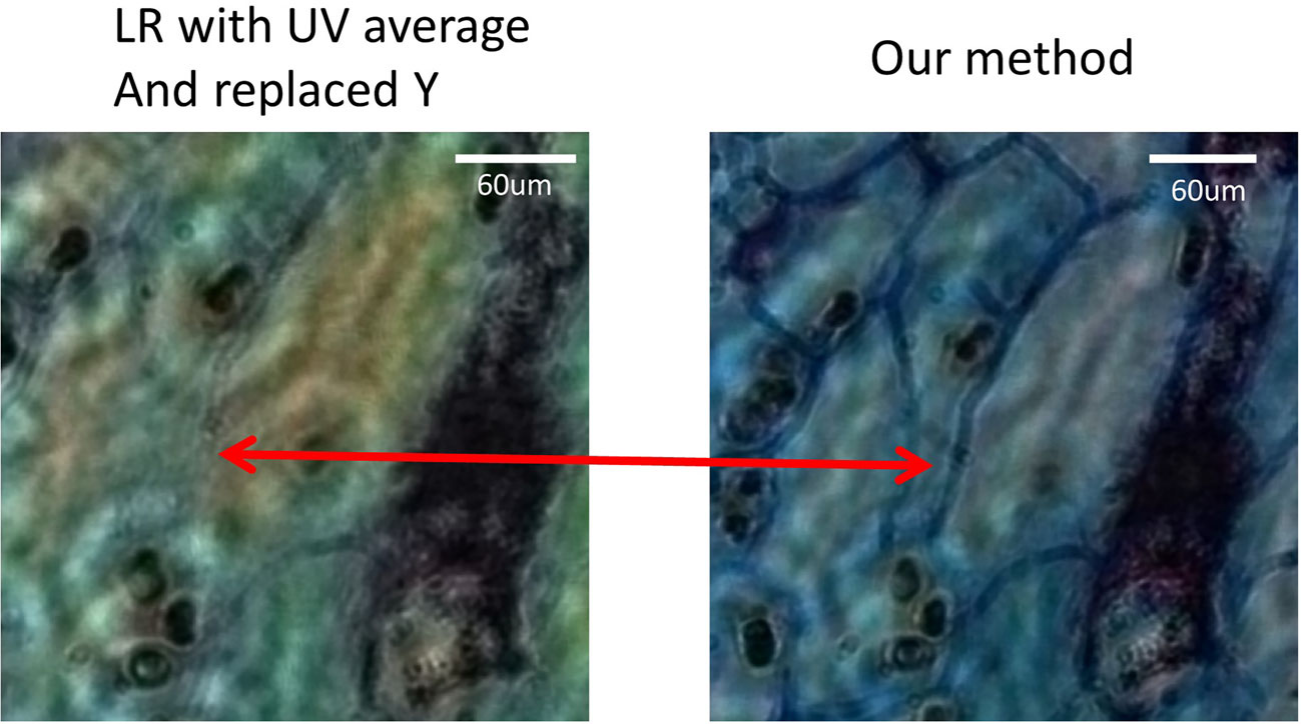
The image of the lotus root cross section proves that only a single band of pixel super-resolution image replaces the Y component, resulting in the loss of certain features.

Since pathological slides are stained to help pathologists to distinguish cell types with significant different colour distribution, the colour information is critical to clinical diagnosis. However, different stained pathological slides have different spectral absorption characteristics. Here, assuming that there are band A and band B for illumination, when the stained pathological slide completely absorbs band A, the transmitted light would be zero, which means there is no response signal detected by the sensor, the image would appear completely black when reconstruction happens in band A. It is considered that if there is material between the light source and the image sensor, the light transmittance of the substance is extremely low. In the same way, when the stained pathological section completely transmits to band B, the absorption would be zero. As a result, the image appears completely white based on the reconstruction in band B, which means that there is no substance between the light source and the image sensor. Since if only a narrow band is used to describe the stained pathological slide, the result will be incomplete and inevitably cause some structural information to get lost. Therefore, if the low-resolution RGB image is converted to the YUV space, the UV space would be utilised for mean filtering, and the Channel Y is replaced with a single-band high-resolution image. Despite the fact that the workload of acquiring image data is reduced, the information loss is more serious. We carried out the experiment by using the lotus root cross section to convert low-resolution RGB image to the YUV space, with UV for averaging, and the Channel Y replaced by a single-band high-resolution image, as well as the high-resolution RGB image being converted to YUV space, with UV being used for mean filtering, without any process in Channel Y. It can be seen from **Figure 6** that our proposed method was better not only in eliminating rainbow colour artifacts but also in terms of texture integrity.

In conclusion, we proposed a high-resolution wavelength scanning colour reconstruction method, which improved the resolution from [group 8, element 2] to [group 8, element 4] and presented satisfactory rainbow-like artifact elimination as well as texture integrity display. It is expected to obtain sub-micron resolution when using CMOS with smaller pixels. By improving the beam quality of the light source, higher-quality colour reconstruction can be expected to appear. Recently, the newly proposed neural network-based method has excellent performance in pixel super-resolution, phase retrieval and colour reconstruction. It can significantly reduce the number of samples without iteration and achieve end-to-end mapping (26, 31). If the method in this paper is combined with a neural network, further improvement in imaging speed and quality can be expected.

## Data Availability Statement

The raw data supporting the conclusions of this article will be made available by the authors, without undue reservation.

## Author Contributions

GZ performed experiments and drafted the manuscript. WQ proposed the idea, made discussions and composed the manuscript together with GZ. JH revised the manuscript. All authors contributed to the article and approved the submitted version.

## Funding

This work was supported by the Ministry of Science and Technology’s key research and development program (No. 2020YFC2003800) and Shenzhen Science and Technology Program of China grant JCYJ20200109115420720.

## Conflict of Interest

The authors declare that the research was conducted in the absence of any commercial or financial relationships that could be construed as a potential conflict of interest.

## Publisher’s Note

All claims expressed in this article are solely those of the authors and do not necessarily represent those of their affiliated organizations, or those of the publisher, the editors and the reviewers. Any product that may be evaluated in this article, or claim that may be made by its manufacturer, is not guaranteed or endorsed by the publisher.
